# Chemoautotrophic growth of ammonia-oxidizing *Thaumarchaeota* enriched from a pelagic redox gradient in the Baltic Sea

**DOI:** 10.3389/fmicb.2014.00786

**Published:** 2015-01-15

**Authors:** Carlo Berg, Luisa Listmann, Verona Vandieken, Angela Vogts, Klaus Jürgens

**Affiliations:** ^1^Biological Oceanography, Leibniz Institute for Baltic Sea Research Warnemünde (IOW)Rostock, Germany; ^2^Paleomicrobiology Group, Institute for Chemistry and Biology of the Marine Environment, Carl von Ossietzky University of OldenburgOldenburg, Germany

**Keywords:** ammonia-oxidizing archaea, Baltic Sea, chemoautotrophy, CO_2_-fixation, enrichment, *Thaumarchaeota*

## Abstract

Ammonia-oxidizing archaea (AOA) are an important component of the planktonic community in aquatic habitats, linking nitrogen and carbon cycles through nitrification and carbon fixation. Therefore, measurements of these processes in culture-based experiments can provide insights into their contributions to energy conservation and biomass production by specific AOA. In this study, by enriching AOA from a brackish, oxygen-depleted water-column in the Landsort Deep, central Baltic Sea, we were able to investigate ammonium oxidation, chemoautotrophy, and growth in seawater batch experiments. The highly enriched culture consisted of up to 97% archaea, with maximal archaeal numbers of 2.9 × 10^7^ cells mL^−1^. Phylogenetic analysis of the 16S rRNA and ammonia monooxygenase subunit A (*amoA*) gene sequences revealed an affiliation with assemblages from low-salinity and freshwater habitats, with *Candidatus* Nitrosoarchaeum limnia as the closest relative. Growth correlated significantly with nitrite production, ammonium consumption, and CO_2_ fixation, which occurred at a ratio of 10 atoms N oxidized per 1 atom C fixed. According to the carbon balance, AOA biomass production can be entirely explained by chemoautotrophy. The cellular carbon content was estimated to be 9 fg C per cell. Single-cell-based ^13^C and ^15^N labeling experiments and analysis by nano-scale secondary ion mass spectrometry provided further evidence that cellular carbon was derived from bicarbonate and that ammonium was taken up by the cells. Our study therefore revealed that growth by an AOA belonging to the genus *Nitrosoarchaeum* can be sustained largely by chemoautotrophy.

## Introduction

The aerobic oxidation of ammonia (NH_3_) or ammonium (NH^+^_4_) to nitrite (NO^−^_2_) is an essential step in the cycling of nitrogen and was long assumed to be performed exclusively by distinct members of *Bacteria*. This paradigm changed (Francis et al., 2007; Lam et al., 2009) following the discovery of the ammonia monooxygenase subunit A (*amoA*) gene among *Archaea* (Venter et al., 2004; Treusch et al., 2005) and the subsequent isolation of the first marine representative of the ammonia-oxidizing archaea (AOA), *Candidatus* Nitrosopumilus maritimus (Könneke et al., 2005). Molecular surveys inferring the presence of AOA via the detection of *amoA* recognized their global prevalence in many marine habitats (e.g., Francis et al., 2005). All known archaea possessing the *amoA* gene affiliate within the novel phylum *Thaumarchaeota* (Brochier-Armanet et al., 2008; Spang et al., 2010), formerly assigned to the *Crenarchaeota*. The global distribution and high abundances of AOA (Francis et al., 2005) point to their major impact on biogeochemical cycles. Cell-specific balances of carbon utilization and ammonia oxidation allow estimates of overall fluxes in specific ecosystems. In field studies, a correlation was determined between archaeal *amoA* transcripts and the occurrence of *Thaumarchaeota* (Caffrey et al., 2007; Beman et al., 2008). As deduced from the growth conditions of isolates (Könneke et al., 2005; Tourna et al., 2011), culture enrichments (e.g., Hatzenpichler et al., 2008; Jung et al., 2011; Matsutani et al., 2011; Santoro and Casciotti, 2011; French et al., 2012; Lebedeva et al., 2013), and the gene sets detected in AOA genomes (e.g., Hallam et al., 2006a,b; Walker et al., 2010; Blainey et al., 2011; Spang et al., 2012), ammonia oxidation serves to conserve energy and the fixation of inorganic carbon to generate biomass. Yet, variations have also been described, such as the utilization of urea (Hallam et al., 2006a,b; Alonso-Sáez et al., 2012) or the absence of ammonia oxidation despite the expression of *amoA* (Mußmann et al., 2011). Additionally, autotrophy as the sole carbon source for growth has been debated for AOA. In a study of wastewater treatment plants, no evidence of CO_2_ fixation by AOA was found, despite their abundance and active growth (Mußmann et al., 2011). Furthermore, the utilization of organic carbon by marine archaeal (Ouverney and Fuhrman, 2000) or thaumarchaeotal assemblages (Teira et al., 2006) as well as by AOA isolates was reported: for example, the growth of *Nitrososphaera viennensis* was substantially enhanced when pyruvate was provided as an additional organic carbon source (Tourna et al., 2011; Stieglmeier et al., 2014), and two recent isolates related to *Ca*. N. maritimus showed obligate mixotrophy, since their growth depended on the assimilation of organic carbon compounds (Qin et al., 2014).

Due to their chemolithoautotrophic lifestyle, in many ecosystems AOA are part of the organismal backbone involved in element transformations. Studies of the genomes of *Ca*. Nitrosopumilus maritimus, *Nitrososphaera viennensis*, and *Ca*. Nitrosoarchaeum limnia, revealed carbon fixation via a modified 3-hydroxypropionate/4-hydroxybutyrate cycle (3-HP/4-HB) (Berg et al., 2007; Walker et al., 2010; Blainey et al., 2011; Tourna et al., 2011). The use of this very cost-effective CO_2_ fixation pathway by AOA (Könneke et al., 2014) distinguishes them from autotrophic ammonia-oxidizing bacteria, which use the Calvin-Benson-Basham cycle (e.g., Chain et al., 2003; Klotz et al., 2006). AOA capable of using the 3-HP/4-HB-cycle may also metabolize small organic substrates, as suggested by Hatzenpichler (2012). Despite the fact that most enriched or isolated *Thaumarchaeota* grow chemoautotrophically on inorganic media, the relationship between ammonia oxidation and chemoautotrophy has not been studied. Direct measurements of CO_2_ fixation by AOA are scarce and the fraction of AOA that live autotrophically is unknown for most environments.

Besides three previously reported marine AOA isolates (Könneke et al., 2005; Qin et al., 2014), AOA enrichment cultures from various sources have been established and investigated, e.g., from freshwater (French et al., 2012), estuarine sediments (Mosier et al., 2012), the ocean (Wuchter et al., 2006; Santoro and Casciotti, 2011), agricultural soil (Jung et al., 2011), and thermal habitats (Hatzenpichler et al., 2008), among others. These studies have contributed new details on the physiology, niche partitioning, and biogeochemistry of *Thaumarchaeota*. Because data on specific biogeochemical activities of AOA in natural environments are difficult to acquire, activity-based experiments with AOA enrichment cultures offer a suitable alternative approach.

In oxygen-depleted waters of the Baltic Sea, *Thaumarchaeota* account for up to one third of the total cell counts and thus constitute a substantial fraction of the microbial community (Labrenz et al., 2010; Berg et al., 2014). At the overlap of oxygen and ammonium gradients, AOA are the main catalyzers of ammonia oxidation (Berg et al., 2014). Moreover, they supply oxidized N for denitrification, a relevant N-loss process in the Baltic Sea nitrogen cycle, that is carried out in pelagic redox gradients mainly by chemoautotrophic epsilonproteobacteria (Grote et al., 2012).

In this study, we investigated the balances of chemoautotrophy and ammonium oxidation in an AOA enrichment culture obtained from the Landsort Deep redox gradient, central Baltic Sea. Our findings provide insights into the coupling between ammonium oxidation and carbon fixation in this enrichment and therefore on the relevance of chemoautotrophy for the generation of biomass by an AOA of the genus *Nitrosoarchaeum*.

## Materials and methods

### Retrieval of environmental samples, enrichment, and cultivation

Water from the Baltic Sea Landsort Deep station TF284 (58° 35.0183N, 018° 14.0795E) was retrieved onboard the *R/V Heincke* in November 2010. Samples were taken from the pelagic redox gradient at a depth of 90 m, with salinity of 10.0 and where oxygen was depleted to 2.5 μmol L^−1^, so that a high abundance of *Thaumarchaeota* can be expected (Labrenz et al., 2010; Berg et al., 2014). After sampling and during enrichment, the water was kept under oxic conditions and in the dark; 1 mmol NH_4_Cl L^−1^ and 50 mg streptomycin L^−1^ were added. The sample bottles were stored at room temperature with aerobic headspace and occasionally screened for NO^−^_2_ production according to the method described by Grasshoff et al. (1983). To select for the typically small thaumarchaeotal cells (diameter <0.22 μm, Könneke et al., 2005; Labrenz et al., 2010), water from nitrite-positive bottles was filtered through 0.45-μm syringe filters and the filtrate further incubated. Enrichments with continuous NO^−^_2_ production and increased archaeal cell numbers were inoculated into 0.1-μm filtered seawater collected from Baltic Sea redox gradients and supplemented with NH_4_Cl and streptomycin, as described above, to further promote the growth of AOA. After approximately 1.5 years, with occasional transfers of 10–15% of the volume into 0.1-μm filtered seawater, archaea numerically dominated the enrichments. A batch growth experiment was carried out, during which growth was monitored along with chemoautotrophy and ammonium oxidation. All batch enrichment cultures were kept in 1-L or 2-L bottles at 22°C in incubation chambers and in the dark. Concentrations of ammonium and nitrite were determined according to the methods of Grasshoff et al. (1983). Ammonium was measured directly after sampling; nitrite samples were filtered through 0.2-μm filters and stored at −20°C until analysis. All batch cultures were inoculated from the same initial enrichment.

### Cell quantification

Depending on the cell density, 0.1-4 mL subsamples of the enrichment cultures were fixed for 2–6 h with particle-free formaldehyde (2% final concentration) and subsequently filtered on 0.2-μm polycarbonate filters (Whatman, 25 mm diameter). Archaeal or bacterial cells on the filter slices were specifically hybridized via catalyzed reporter deposition fluorescence *in situ* hybridization (CARD-FISH) according to Pernthaler et al. (2002), using either the Arc915 probe targeting archaea (Stahl and Amann, 1991) or the EUB338/EUB338II-III probe mix targeting bacteria (Amann et al., 1990; Daims et al., 1999). Cells on the hybridized filters were counter-stained with 4′,6-diamidino-2-phenylindol (DAPI) in Vectashield mounting medium (Vector Labs, Burlingame, California, USA). Ten microscopic fields were randomly selected to count DAPI-stained and specifically hybridized cells using a Zeiss Axioskop 2 mot plus epifluorescence microscope (Zeiss, Oberkochen, Germany). For three of the enrichment cultures (A, B, and C), filters were prepared and analyzed in triplicate; cells that were hybridized during CARD-FISH were counted on slices from one filter.

### CO_2_ fixation rates

Subsamples of 2–4 mL were taken from the batch cultures and incubated in triplicate together with a killed control (fixed with 2% formaldehyde) for 6–24 h after the addition of 0.56–1.85 MBq of NaH^14^CO_3_ (Hartmann Analytic GmbH, Germany), depending on the cell density. The incubations were stopped by filtration onto 0.2-μm polycarbonate (Whatman, 25 mm diameter) filters. Prior to filtration, 50 μL were withdrawn to determine the total radioactivity added to each vial. The filters were exposed to HCl fumes for 0.5–2 h and then transferred into 4 mL of LumaSafe scintillation cocktail (PerkinElmer). Total- and filter-^14^C disintegrations per minute were analyzed with a PerkinElmer Tri-Carb 2800R liquid scintillation analyzer. CO_2_ fixation rates were derived from the fraction of ^14^C incorporated in relation to the total activity added and taking into account the dissolved inorganic carbon concentration of 2 mmol L^−1^ that is characteristic of Baltic Sea redox gradients (Grote et al., 2008; Berg et al., 2014). CO_2_ fixation rates were calculated as follows:

$${\text{CO}}_{2}\text{ fixation}=\frac{\frac{dpm_{f}-dpm_{d}}{dpm_{l}}\times DIC}{t}$$

where *t* is the incubation time; *dpm_f_*, the filter disintegrations per minute; *dpm_d_*, the dpm counts on filters of the killed control; *dpm_l_*, the dpm counts in the liquid sample; and DIC, the ambient concentration of dissolved inorganic carbon.

Mean CO_2_ fixation rates and the increase in cell numbers during the exponential growth phase (from *t*_1_ to *t*_2_) between days 19 and 32 (enrichments A, B, and C) and days 32 and 54 (enrichment E) were considered in calculating the carbon content of one cell as follows:

$$C_{content}=\frac{\frac{{\left[{\text{CO}}_{2}\text{ fixation rate}\right]}_{t_{2}}+{\left[{\text{CO}}_{2}\text{ fixation rate}\right]}_{t_{1}}}{2}\times \left(t_{2}-t_{1}\right)}{{\left[\text{cells }mL^{-1}\right]}_{t_{2}}-{\left[\text{cells }mL^{-1}\right]}_{t_{1}}}$$

To determine the accumulated amount of fixed CO_2_, the fixation rates were multiplied by the number of days until the next CO_2_ fixation rate measurement. These intervals were then cumulatively added for the specific time points of the CO_2_ fixation rate measurements. A correlation was tested by linear bivariate regression using the PAST software package v3.0 (Hammer et al., 2001).

### Uptake of ^13^C-bicarbonate and ^15^N-ammonium and NanoSIMS imaging

Enrichment culture G was amended with 2 mmol NaH^13^CO_3_ L^−1^ (99% ^13^C, Cambridge Isotope Laboratories, Massachusetts, USA) directly after inoculation; 58 μmol ^15^NH_4_Cl L^−1^ (99% ^15^N, Cambridge Isotope Laboratories, Tewksbury, Massachusetts, USA) was added during the exponential growth phase (on day 40). For single-cell analysis by nano-scale secondary ion mass spectrometry (NanoSIMS) (e.g., Musat et al., 2012), samples from the enrichment were taken at several time points and processed using the same procedure described for CARD-FISH except that they were filtered onto gold-coated 0.2-μm polycarbonate filters. The filter surface was gold-sputter-coated with an Agar sputter coater (model 108) for 120 s, resulting in a 20–40-nm thin layer of gold. Incorporation of the label into single cells was assessed using a Cameca NanoSIMS 50L, by measuring the secondary ions emitted in response to sputtering selected areas of the filter surface by a Cs^+^ primary ion beam. The primary ion beam current was 2 pA, with scanning parameters of 256 × 256 pixels for areas of 20 × 20–30 × 30 μm, with a dwell time of 1 μs per pixel. The mass resolving power was adjusted to suppress interferences at all masses. Data of the secondary ion counts were analyzed using the MATLAB R2011b (The MathWorks, USA) based Look@NanoSIMS software (Polerecky et al., 2012). Thus, 30–60 planes were aligned and included in the analysis; regions of interest were defined using the biomass signal based on the ^12^C^14^N- counts. Ratios of ^13^C/^12^C were derived from the secondary ion counts of ^13^C^−^ and ^12^C^−^, respectively, to determine ^13^C enrichment. Cellular ^15^N uptake was determined by calculating the ratio ^15^N^12^C-/^14^N^12^C-.

### DNA-extraction and phylogenetic analysis of the 16S rRNA and *amoA* genes

Samples were taken on day 54, i.e., during the late exponential phase, from enrichment cultures D–G and filtered on 0.2-μm GVWP filters (Millipore). DNA was extracted as described in Weinbauer et al. (2002). DNA of the nearly full-length 16S rRNA gene was PCR-amplified using the Fermentas *taq* polymerase and the primer pairs Arch21f/1492r (Lane, 1991; DeLong, 1992) for *Archaea* and 27f/1492r (Lane, 1991) for *Bacteria*. The archaeal *amoA* primer pairs were Arch-amoAF/Arch-amoAR (Francis et al., 2005); the beta- and gammaproteobacterial *amoA* primers were *amoA*-1F/2R and *amoA*-3F/4R, respectively (Purkhold et al., 2000). PCR products were purified using the Agencourt AMPure kit XP (Beckman Coulter, Krefeld, Germany) according to the manufacturer's instructions. Both the 16S rRNA gene and the *amoA* gene amplicons were cloned with the StrataClone PCR cloning kit (Agilent, Karlsruhe, Germany) as described by the manufacturer and sequenced by LGC Genomics (Berlin, Germany). The sequences were quality-revised with DNAStar SeqMan II v5.06 and forward and reverse sequences were assembled into a contig. Chimeric sequences were detected using DECIPHER (Wright et al., 2012). Phylogenetic analysis was conducted using the ARB 5.1 software package (Ludwig et al., 2004). The 16S rRNA gene sequences were aligned with the online SILVA incremental aligner (SINA) v1.2.11 (Pruesse et al., 2012), then imported into ARB and inspected for alignment errors. Translated *amoA* protein sequences were aligned using the *amoA* ARB database provided by Pester et al. (2012). The 16S rRNA gene phylogenetic tree was constructed with the Phylip neighbor-joining algorithm, Jukes-Cantor correction, and using the ssuref:archaea filter provided in the SILVA SSU Ref NR 99 ARB database (Quast et al., 2013). The *amoA* neighbor-joining tree was generated based on the protein alignment, using Phylip with a Fitch model and *Ca*. N. maritimus SCM1 as a filter. The *amoA* gene and 16S rRNA gene sequences were deposited in the European Nucleotide Archive under the accession numbers LN590601–LN590650 and LN590651–LN590669, respectively.

## Results

### Enrichment of ammonia-oxidizing archaea

**Figure 1:**
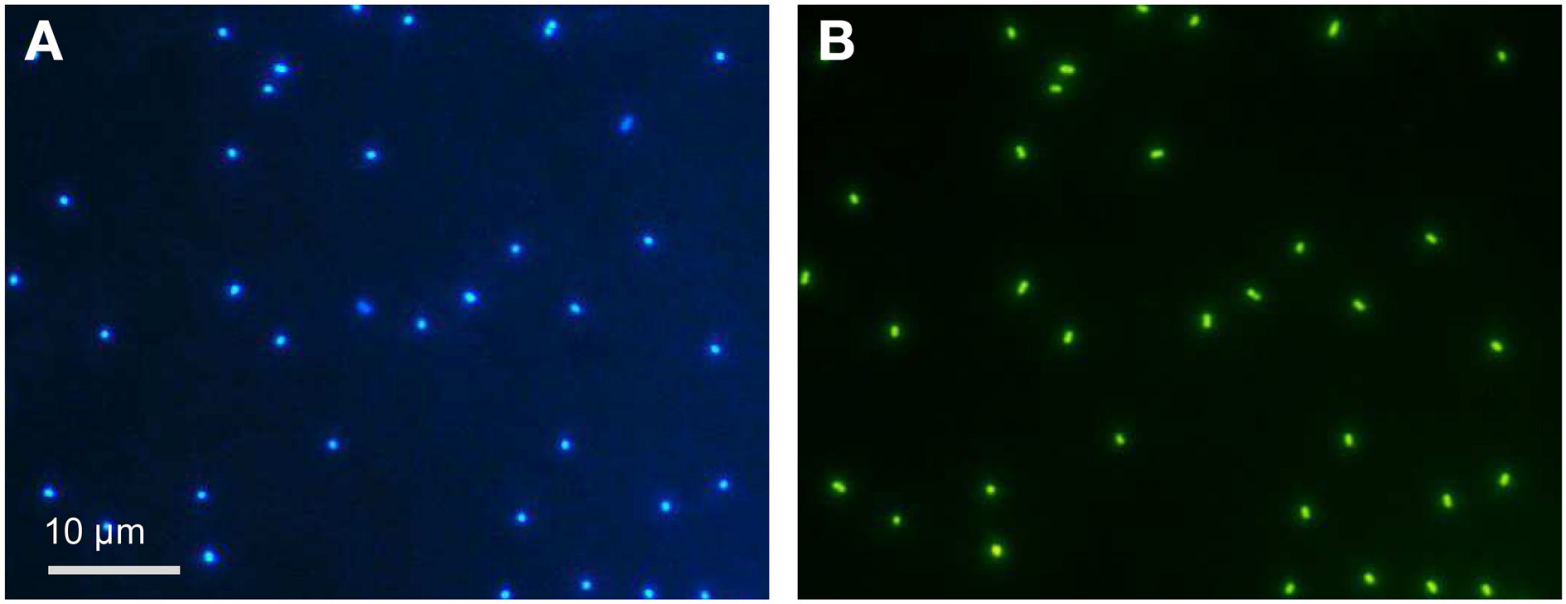
**Epifluorescence microscopy of representative cells from enrichment culture B on day 1**. The cells were stained with DAPI **(A)** or hybridized with the archaea-specific probe Arc915 **(B)** and then analyzed by CARD-FISH. Same field of view; the scale bar represents 10 μm.

**Figure 2:**
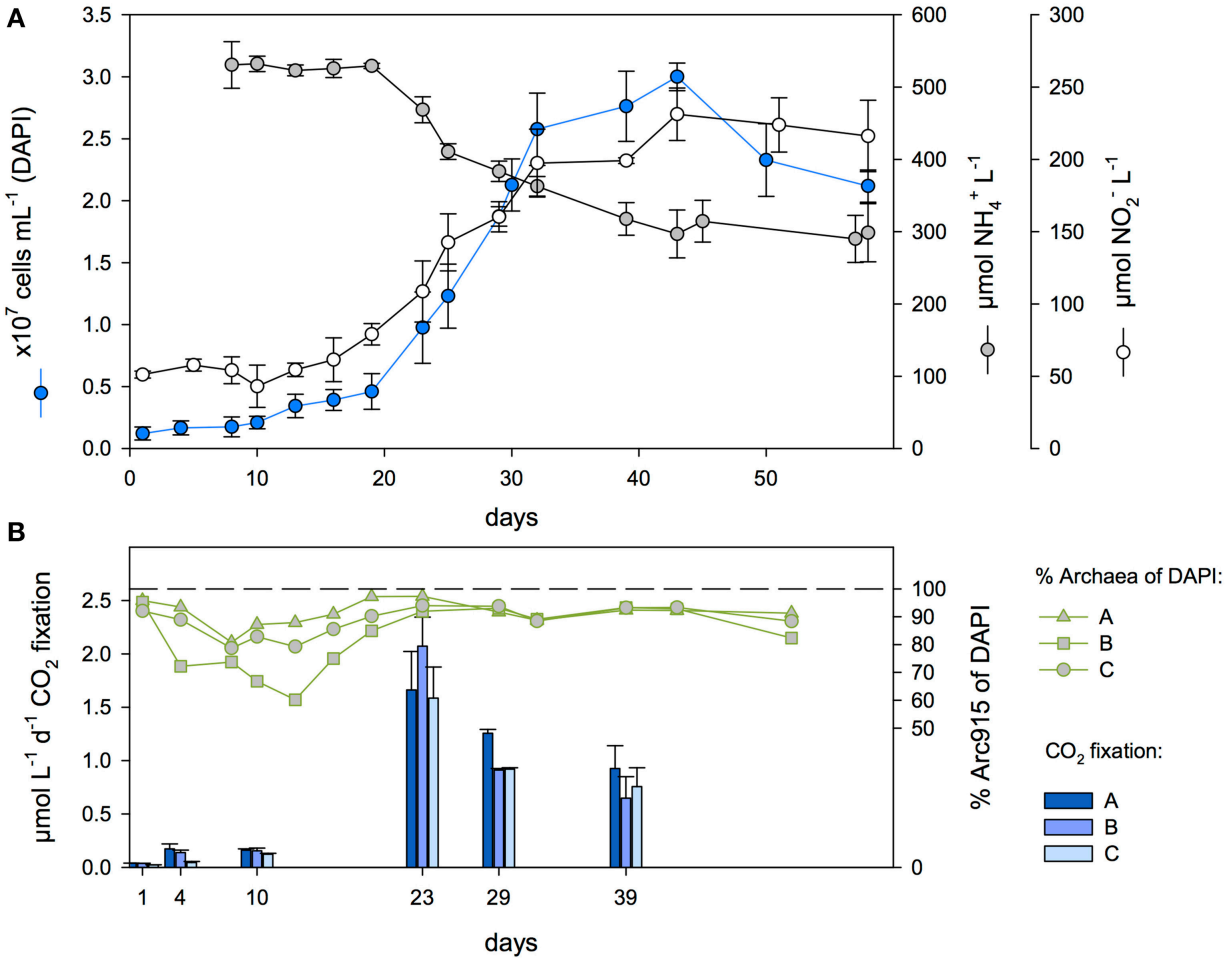
**Cell numbers, ammonium and nitrite concentrations (A) as well as CO_2_ fixation rates, and the archaeal fraction (B) in triplicate enrichment cultures (termed A, B and C)**. Error bars show the standard deviations of cell counts and nitrite and ammonium concentrations from cultures grown in triplicate.

Samples obtained from oxygen-depleted waters of the Baltic Sea were incubated for 1.5 years under conditions promoting the growth of AOA and resulted in cultures highly enriched in AOA (Figure 1). AOA enrichment was evident from nitrite production, the consumption of ammonium, and the concurrent increase in archaeal cell numbers (Figure 2). CARD-FISH for archaea revealed that cells similar in size and morphology to *Ca*. N. maritimus (Könneke et al., 2005; Figure 1) dominated the cultures.

**Figure 3:**
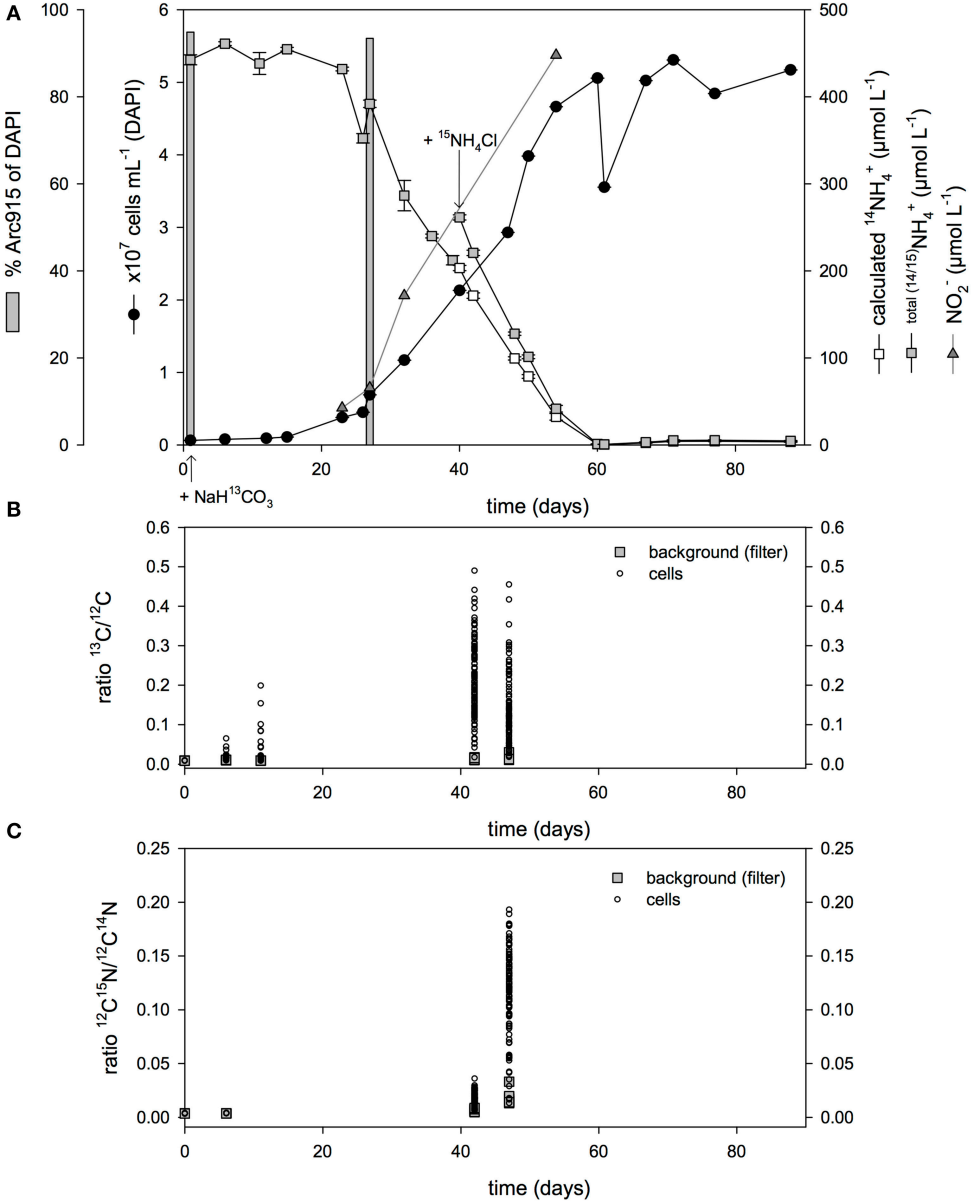
**(A)** Growth curve of enrichment culture G, showing ammonium consumption, total cell numbers, and the fraction of archaeal cells. ^15^N-labeled ammonium was added on day 40. The combined ^14^NH^+^_4_ and ^15^NH^+^_4_ concentration is plotted together with the calculated concentration of ^14^NH^+^_4_ based on the percentage of labeling. Ratios of ^13^C vs. ^12^C **(B)** and ^15^N vs. ^14^N **(C)** enrichment in single cells was determined using NanoSIMS. Error bars show the standard deviation among triplicate samples. The data point of day 0 corresponds to unlabeled cells from enrichment E.

In a batch growth experiment at 22°C, total cell numbers during exponential growth reached a maximum of 3.1 × 10^7^ cells mL^−1^, with archaeal cells comprising 93–97%, as determined by CARD-FISH (Figure 2), and bacterial cells, identified with the probes EUB338I-III on day 43, 5–10% (cultures A, B, and C). After a lag phase of 19 days, the archaeal cells grew exponentially, with a generation time of 4.5 days (Table 1). Ammonium consumption during exponential growth accounted for an oxidation rate of 12.81 ± 0.80 μmol NH^+^_4_ L^−1^ d^−1^ and was accompanied by the increase in archaeal cell numbers and the near-stoichiometric accumulation of nitrite at a rate of 9.42 ± 1.53 μmol L^−1^ d^−1.^ Culture G showed similar properties but with a longer generation time (Table 1): total cell numbers reached 5.2 × 10^7^ cells mL^−1^ and ammonium was completely consumed at a rate of 11.55 μmol NH^+^_4_ L^−1^ d^−1^, comparable to that by cultures A–C (Figure 3). Enrichment cultures D and F (Figure A2) grew more slowly and less continuously, reaching maximum total cell abundances of 1.5×10^7^ cells mL^−1^. The growth of enrichment culture E (Figure A2) was similar to that of cultures A–C and G.

**Table 1:** **Growth characteristics and balances during the exponential phase of enrichment cultures grown on natural 0.1-μm filtered seawater**.

**Parameter**	**Cultures**					**Unit**
	**A, B, C (mean ± SD)**	**D**	**E**	**F**	**G**	
NH^+^_4_ oxidation	12.81 ± 0.80	5.31	12.32	6.99	11.55	μmol L^−1^ d^−1^
NO^−^_2_ accumulation	9.42 ± 1.53	-	-	-	18.99	μmol L^−1^ d^−1^
CO_2_ fixation (bulk)	1.28 ± 0.10	0.31	1.12	0.17	-	μmol L^−1^ d^−1^
CO_2_ fixation (max., Arc915 cell-specific)	0.2 ± 0.06	-	-	-	-	fmol C cell^−1^ d^−1^
C-content cell^−1^ (Arc915)	9.02 ± 0.46	-	-	-	-	fg C cell^−1^
C-content cell^−1^ (DAPI)	9.42 ± 0.67	42.99	13.28	13.04	-	fg C cell^−1^
Generation time (Arc915)	4.47 ± 0.65	-	-	-	-	days
Generation time (DAPI)	5.20 ± 0.63	94.25	12.78	21.75	11.03	days
N oxidized per C incorporated	10.07 ± 0.81	16.86	11.03	41.25	-	Ratio

*Ammonium oxidation rates were determined by measuring the decrease in NH^+^_4_ concentrations. -, not determined; SD, standard deviation of triplicate cultures*.

### CO_2_ fixation

**Figure 4:**
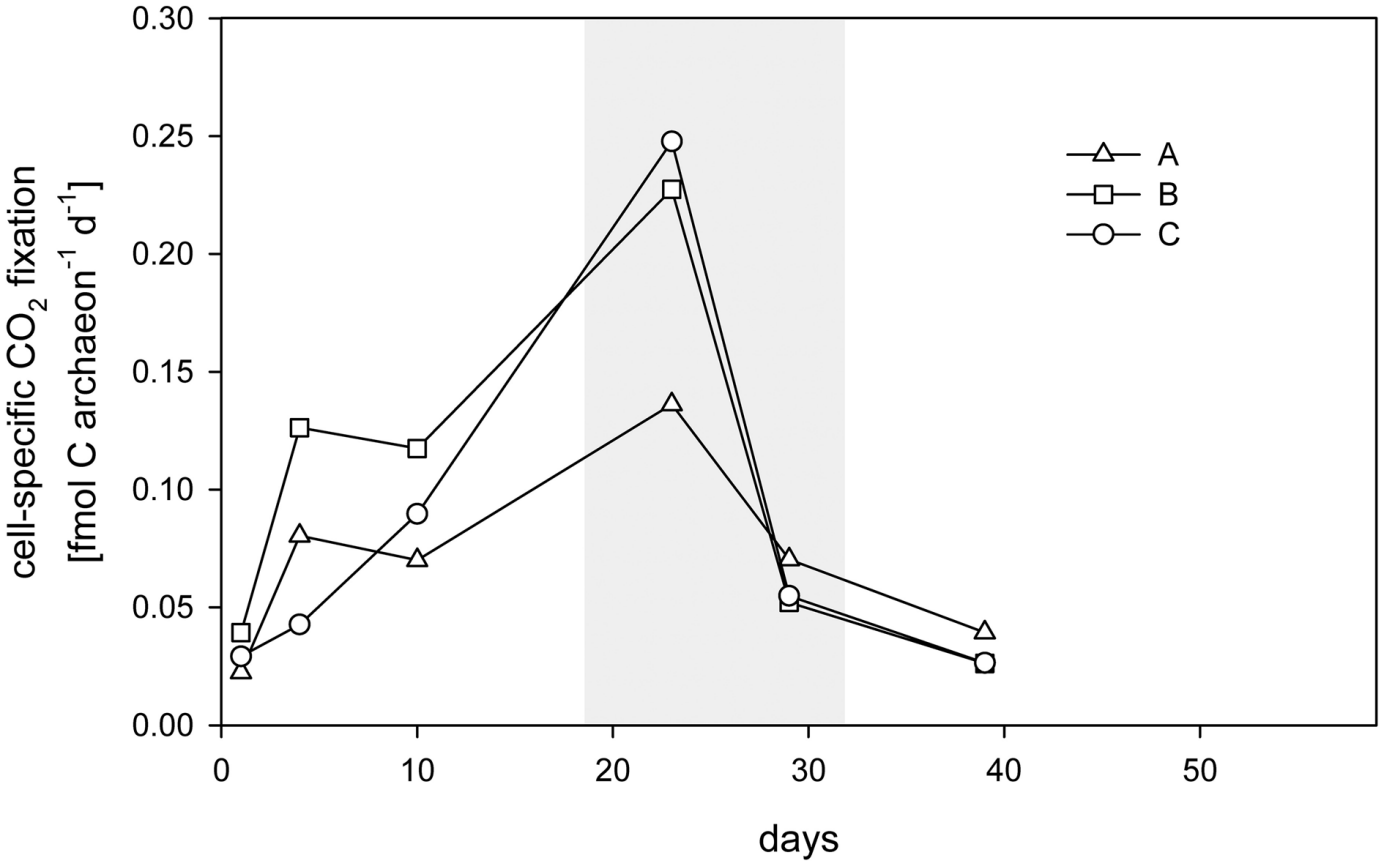
**Cell-specific CO_2_ fixation rates calculated from archaeal cell numbers and bulk CO_2_ fixation rates during the growth of enrichment cultures A, B, and C**. The exponential phase is shown in gray.

CO_2_ fixation rates in cultures A, B, and C reached 161 nmol L^−1^ d^−1^ (Figure 2B) during the lag phase and, together with cell numbers and ammonium consumption, increased substantially with the onset of the exponential growth phase. Bulk rates of up to 1585–2073 nmol C L^−1^ d^−1^ were determined at early exponential phase, declining to 648–926 nmol C L^−1^ d^−1^ during early stationary phase. Calculated archaeal-cell-specific CO_2_ fixation rates were highest on day 23, at 0.2 ± 0.06 fmol C cell^−1^ (Table 1 and Figure 4). Determination of the cumulative amount of CO_2_ fixed over time showed that the total amount of incorporated C correlated with the increase in Arc915-hybridized cell counts (e.g., culture A: *r*^2^ = 0.992, *p* = 2.4 × 10^−5;^ Figure A1). Based on a comparison of CO_2_ fixation and ammonium oxidation rates in cultures A-C during exponential growth, one atom C was fixed per 10 atoms N oxidized (Table 1).

### Cellular uptake of ^13^C-bicarbonate and ^15^N-ammonium

Enrichment culture G was amended after inoculation with 2 mmol of ^13^C-labeled bicarbonate L^−1^ (Figure 3A), corresponding to 50% ^13^C-labeled bicarbonate, in addition to an ambient concentration of 2 mmol L^−1^ bicarbonate in the seawater-based medium. During the lag phase, the enrichment of ^13^C in individual cells inspected via NanoSIMS increased above the natural ratio of 0.011 (Nier, 1950; International Atomic Energy Agency, IAEA) to 0.2 (Figure 3B and Figure A3). During exponential growth, on days 42 and 47, the ratio of ^13^C/^12^C increased up to 0.5. Among all cells investigated (*n* = 268) the ratios varied; on days 42 and 47, mean ^13^C enrichment was 0.22 ± 0.09 and 0.14 ± 0.09, respectively. NanoSIMS image analysis showed that the labeled cells were analogous in size and morphology to the Arc915-hybridized cells detected via epifluorescence microscopy (Figure 1). Nearly all of the investigated cells were ^13^C-enriched, with enrichment levels above the background values of the polycarbonate filters, the unlabeled cells of the control, and the natural ^13^C/^12^C ratio.

On day 40, ^15^N-labeled ammonium was added, corresponding to 22% ^15^N labeling of the ammonium pool on that day. Subsequently, the ratio of ^15^N vs. ^14^N in the cells was 0.04 on day 42 and as high as 0.19 on day 47, with means of 0.02 ± 0.006 and 0.12 ± 0.04, respectively (Figure 3D and Figure A4). These ratios were considerably higher than the natural ratio of 0.004 (Nier, 1950).

### Phylogenetic affiliation

**Figure 5:**
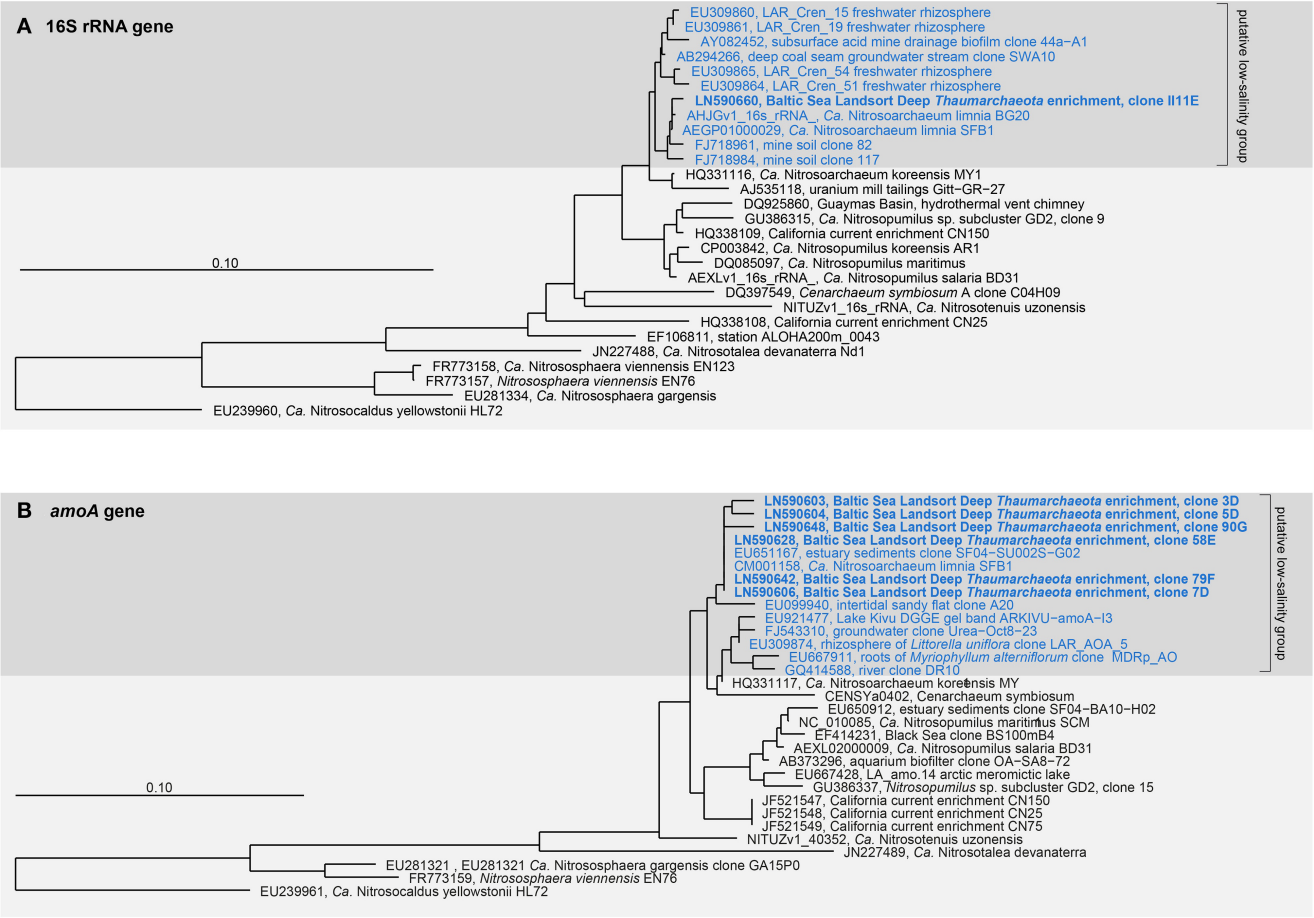
**Neighbor-joining trees showing the phylogenetic placement of the enrichment based on the cloned, nearly full-length 16S rRNA gene (A) and *amoA* gene (B) sequences**. The scale bar represents 10 iterations per 100 nucleotides **(A)** or amino acids **(B)**.

Amplification of the bacterial and archaeal 16S rRNA genes from DNA extracts of the enrichment cultures yielded products only for *Archaea*. All 16S rRNA gene sequences (*n* = 19) thus obtained fell within the *Nitrosopumilus* cluster and were closely related to each other. The closest phylogenetic relative was *Ca*. Nitrosoarchaeum limnia SFB1 (NZ_CM001158.1), with 98.8–99.9% 16S rRNA gene sequence identity (Figure 5A). No beta- or gammaproteobacterial *amoA* gene sequences were amplified, whereas all archaeal *amoA* gene sequences (*n* = 50) were affiliated with a putative low-salinity group (Figure 5B; Blainey et al., 2011). With respect to their *amoA* gene sequences, the enrichment had 99.4–99.7% sequence identity with *Ca*. N. limnia SFB1 (NZ_CM001158.1).

## Discussion

Ammonia-oxidizing *Thaumarchaeota* may be among the most relevant chemolithoautotrophs in aquatic habitats. Our enrichment of AOA (up to 97% purity, Figures 1, 2) of the genus *Nitrosoarchaeum* showed that in seawater-based medium inorganic carbon fixation was sufficient to generate all cellular carbon. Ammonium utilization was ten-fold higher than dark carbon fixation, providing a relationship between these two processes, which can be carried out by most AOA. Phylogenetically, the enrichment was determined to fall within a putative low-salinity and freshwater group, concurrent with its environmental origin and the intermediate salinities of Baltic Sea redox gradients.

### Autotrophy and carbon content per cell

Growth of the AOA enrichment was accompanied by CO_2_ fixation activity, which substantially increased with the onset of the exponential growth phase (Figure 2). Thus, the major fraction of CO_2_ fixation can be attributed to archaeal growth; only minimal CO_2_ fixation may have been carried out by for example nitrite-oxidizing bacteria, as the presence of streptomycin suppressed bacterial activities, archaea were highly enriched and nitrite accumulated near-stoichiometrically relative to ammonium oxidation. By relating the amount of incorporated inorganic carbon to the increase in archaeal cell numbers during exponential phase we calculated an average of 9.02 fg of chemoautotrophy-derived carbon per newly produced cell (Table 1). This amount is comparable to the 8.39 fg C cell^−1^ reported for autotrophic archaea in the deep Atlantic Ocean (Herndl et al., 2005). By contrast, the mean carbon content of planktonic prokaryotes is about 20 fg C cell^−1^ (Lee and Fuhrman, 1987). However, due to the small cell size of AOA, lower carbon content is expected. Moreover, it suggests that CO_2_ fixation provided the major part of carbon for the production of an archaeal cell, while incorporation of organic carbon may still have occurred, however, at a smaller extent. Notably, this relationship may vary in environmental AOA populations.

The maximal mean cell-specific CO_2_ fixation rate of 0.2 ± 0.06 fmol C cell^−1^ day^−1^ (Figure 4) is higher than the rate calculated for marine archaea in the North Atlantic by Herndl et al. (2005) or Varela et al. (2011), who reported maximum rates of 0.014 and 0.1 fmol C cell^−1^ day^−1^, respectively. These discrepancies may reflect the differences in physicochemical parameters, such as temperature or substrate availability or competition for substrates in natural environments compared to the growth conditions of the enrichment cultures.

Ideally, our calculations would be complemented by measurements of the particulate organic carbon (POC) content in the enrichment cultures, but the low cell concentration and the small cell size did not yield sufficient biomass to allow conventional POC measurements. Attempts to filter the cells onto glass-fiber GF/F filters were not successful because most of the cells passed through them. However, the amount of incorporated carbon that accumulated, based on CO_2_ fixation rates (Figure A1), correlated with the increase in archaeal cell counts during batch growth, likewise suggesting that growth is mainly based on chemoautotrophy. Additionally, NanoSIMS analyses of single cells revealed that, until the exponential phase, up to 50% of the cellular ^13^C carbon originated from labeled bicarbonate (Figures 3B,C), which reflects the fraction of ^13^C-labeled bicarbonate present in the medium. Although the cells analyzed by NanoSIMS were not re-identified by FISH, the dominance of archaea in the enrichment culture (Figure 3A) implies a high probability that the investigated cells were AOA. The enrichment cultures were also susceptible to the archaea-specific biosynthesis inhibitor *N*^1^-guanyl-1,7-diaminoheptane (GC_7_) (Jansson et al., 2000), which led to a substantial decrease in CO_2_ fixation activity (Berg et al., 2014). Taken together, our data show that the generation of the major portion of archaeal biomass in the *Nitrosoarchaeum* enrichment relied on chemoautotrophy. Yet, our approach does not exclude the additional incorporation of small amounts of organic compounds, thus mixotrophic growth, since natural seawater is not free of dissolved organic carbon. For example, *N. viennensis* and the mixotrophic strains HCA1 and PS0, which are related to *Ca*. N. maritimus SCM1, require pyruvate (Tourna et al., 2011) and α-ketoglutaric acid (Qin et al., 2014), respectively. Nonetheless, our results underline the potential role of AOA in the carbon cycle, and their presence may imply preceding contributions to planktonic biomass via chemoautotrophy, especially considering the worldwide distribution of AOA (Francis et al., 2005), presumably dominating the large fraction of pelagic archaea in the dark ocean (Karner et al., 2001).

The shortest generation time in our enrichment was 4.5 days (Table 1), which is substantially longer than the 21–26 h (at 28°C) reported for *Ca*. Nitrosopumilus maritimus SCM1 (Könneke et al., 2005; Martens-Habbena et al., 2009) or the 45 h (at 37°C) of *Nitrososphaera viennensis* (Tourna et al., 2011) but comparable to the 4–4.6 days (at 22°C) determined in AOA enrichment cultures of the California Current that were grown in natural seawater-based medium (Santoro and Casciotti, 2011) and somewhat slower than the 3.4 days of the closest relative, *Ca*. Nitrosoarchaeum limnia SFB1, grown at 22°C (Mosier et al., 2012). These differences may be the result of variations in media composition and cultivation temperatures, with faster growth promoted by warmer conditions. Clearly, the conditions used to obtain the enrichment cultures were different from the natural environment of the Baltic Sea redox gradients, where temperatures in the suboxic zone are year-round 5–7°C. In the latter setting, growth is expected to be slower and may be limited by substrate availability, as maximum *in situ* ammonium oxidation rates are at least 15-fold lower (122–884 nmol N L^−1^ d^−1^) (Berg et al., 2014).

### Coupling between ammonia oxidation and CO_2_ fixation

In the coupling between nitrogen and carbon cycles, AOA play a crucial role. Their high substrate affinity for ammonia (Martens-Habbena et al., 2009) may explain their occurrence in areas of low ammonium concentrations in the open ocean (reviewed by Erguder et al., 2009) and thus their broad distribution. Therefore, the availability of ammonia likely limits biomass production by AOA. In our study, ammonium oxidation and inorganic carbon fixation occurred at a ratio of 10 N oxidized per 1 C incorporated (Table 1), if considering the energetically most efficient ratio (Table 1). To what extent this ratio may vary in natural populations for individual cells remains to be determined. However, it is in line with the 10:1 ratio previously discussed for nitrifiers (Tijhuis et al., 1993; Wuchter et al., 2006; Middelburg, 2011) and also comparable to the bulk nitrification and CO_2_ fixation rates determined in the central Baltic Sea (Berg et al., 2014): In the pelagic redox zone, where the gradients of oxygen and ammonium overlap and *Thaumarchaeota* are present in high numbers, nitrification rates (122–884 nmol N L^−1^ d^−1^) are approximately one order of magnitude higher than those of CO_2_ fixation (19–58 nmol C L^−1^ d^−1^). This suggests that CO_2_ fixation in oxygen-deficient waters of the Baltic Sea is largely mediated by AOA, since other chemoautotrophic microorganisms, such as the gammaproteobacterial SUP05 (Glaubitz et al., 2013) and epsilonproteobacterial *Sulfurimonas* sp., reside and fix CO_2_ in deeper waters of the Baltic redox gradients, mainly around the oxic-anoxic interface and below (Grote et al., 2008).

### Phylogenetic affiliation with a putative low-salinity group

Based on both 16S rRNA gene and *amoA* gene sequences, our Baltic Sea enrichment affiliates most closely with *Ca*. Nitrosoarchaeum limnia (Figure 5) whereas 16S rRNA gene and *amoA* gene sequence identities to the thaumarchaeotal subcluster GD2, which is abundant in the Baltic Sea redox gradients (Labrenz et al., 2010), account for 94.1–98.0% and 89.7–90.1%, respectively.

Many *Thaumarchaeota* sequences recovered from similar habitats cluster in specific groups, indicating that niche partitioning is also reflected in *amoA* sequence diversity. A putative “low-salinity group” comprising sequences collected from streams, estuaries, and groundwater habitats was proposed by Blainey et al. (2011) and by Biller et al. (2012). The salinities characteristic of brackish pelagic redox gradients in the central Baltic Sea are in the range of 6–11 (Labrenz et al., 2007). Sequences recovered from our enrichment culture had 98.8–99.9% 16S rRNA gene sequence identity with *Ca*. Nitrosoarchaeum limnia SFB1 (Blainey et al., 2011), which originated from low-salinity (7.9) sediments of San Francisco Bay (Mosier et al., 2012). Although the two low-salinity habitats are geographically far apart, the sequence identity between *Ca*. N. limnia SFB1 and our enrichment is remarkably high for both the 16S rRNA and the *amoA* genes. Bouskill et al. (2012) argued that the geochemical properties of a habitat strongly determine the distribution of phylogenetic groups of ammonia oxidizers. This conclusion gained support from a comprehensive analysis of *amoA* sequences from aquatic habitats, in which Biller et al. (2012) found that salinity, among other parameters, determined the distribution of niche-specific *amoA* sequence types. Genome sequencing of the enrichment may reveal physiological differences with *Ca*. N. limnia SFB1, as even closely related AOA can inhabit strikingly different ecological niches (Qin et al., 2014).

Our growth experiment with a Baltic Sea AOA enrichment culture extends current knowledge on chemoautotrophic AOA by providing direct evidence that the biomass needed for cell growth is largely generated by the fixation of inorganic carbon. Furthermore, we were able to relate ammonium oxidation to CO_2_ fixation, which has implications in the AOA-mediated coupling of N and C cycles in the environment and may contribute to the modeling of carbon and nitrogen cycles in AOA-dominated habitats.

## Author contributions

Carlo Berg and Verona Vandieken initiated the enrichment cultures. Carlo Berg, Luisa Listmann and Klaus Jürgens designed the growth experiments. Carlo Berg and Luisa Listmann performed experiments and analysis. Angela Vogts performed measurements via NanoSIMS. Carlo Berg wrote the manuscript. Verona Vandieken, Angela Vogts, Luisa Listmann and Klaus Jürgens did proof-reading of the manuscript.

### Conflict of interest statement

The authors declare that the research was conducted in the absence of any commercial or financial relationships that could be construed as a potential conflict of interest.
