# Supplementary material for: Chemoautotrophic growth of ammonia-oxidizing Thaumarchaeota enriched from a pelagic redox gradient in the Baltic Sea
Source: Front Microbiol. 2015 Jan 15;5:786. doi: 10.3389/fmicb.2014.00786 (PMC4295551; doi:10.3389/fmicb.2014.00786)

Supplementary Material

Figure A1

Total amount of carbon fixed over time in three parallel batch cultures (A–C) based on CO_2 fixation rates (see Figure 2B) versus the increase in archaeal cell numbers as determined using CARD-FISH and probe Arc915.

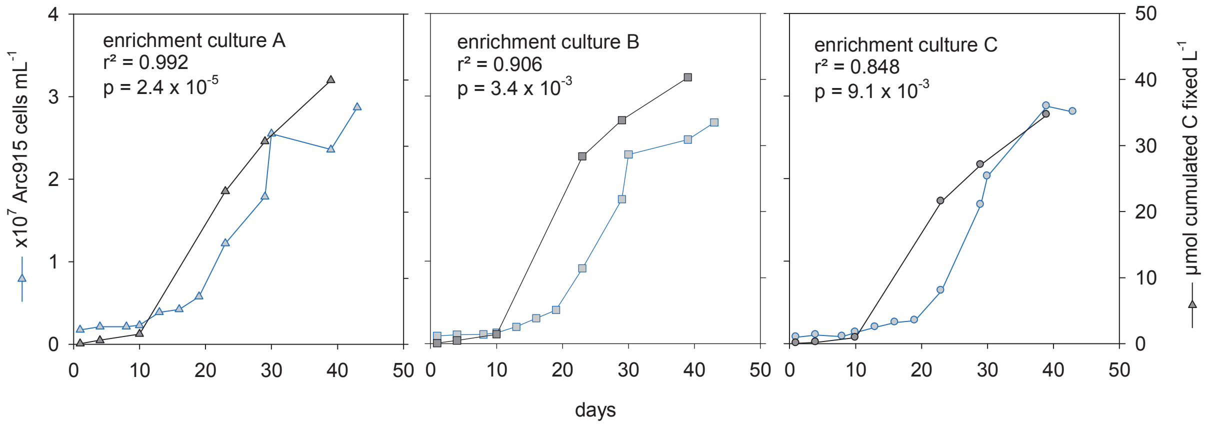

Supplement: Supplementary file 1 [file Image1.PDF]
