# Supplementary material for: Chemoautotrophic growth of ammonia-oxidizing Thaumarchaeota enriched from a pelagic redox gradient in the Baltic Sea
Source: Front Microbiol. 2015 Jan 15;5:786. doi: 10.3389/fmicb.2014.00786 (PMC4295551; doi:10.3389/fmicb.2014.00786)

Figure A2

Ammonium consumption and total cell counts (DAPI) during the batch growth of cultures D, E, and F.

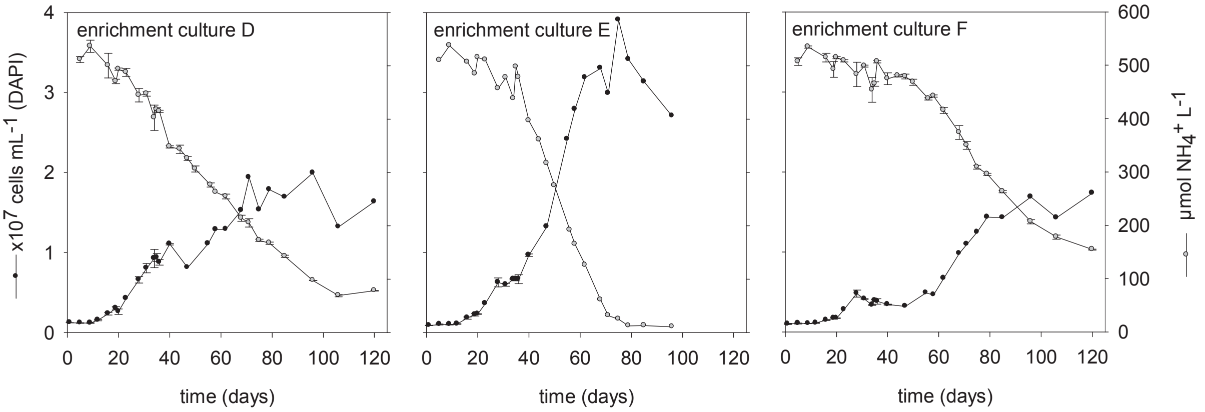

Supplement: Supplementary file 2 [file Image2.PDF]
