# Supplementary material for: Chemoautotrophic growth of ammonia-oxidizing Thaumarchaeota enriched from a pelagic redox gradient in the Baltic Sea
Source: Front Microbiol. 2015 Jan 15;5:786. doi: 10.3389/fmicb.2014.00786 (PMC4295551; doi:10.3389/fmicb.2014.00786)

Figure A3

Nano-scale secondary ion mass spectrometry (NanoSIMS) images of ^{13}C-enriched cells sampled 11 days after inoculation and amendment with 2 mmol ^{13}C-bicarbonate L^{-1}. Left: $^{12}C^{14}N^{-}$ counts representing organic material, right: $^{13}C/^{12}C$ ratio representation derived from pixel by pixel calculations from the $^{12}C^{-}$ and $^{13}C^{-}$ signal counts.

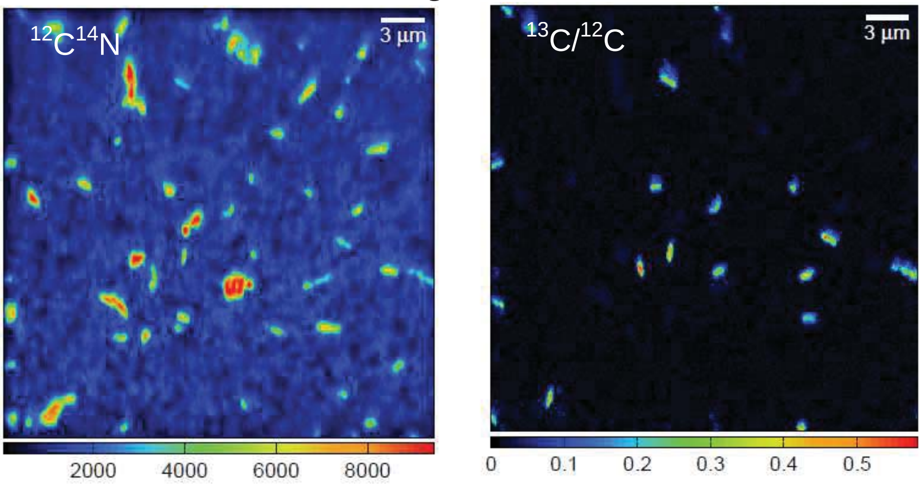

Supplement: Supplementary file 3 [file Image3.PDF]
