# Supplementary material for: Chemoautotrophic growth of ammonia-oxidizing Thaumarchaeota enriched from a pelagic redox gradient in the Baltic Sea
Source: Front Microbiol. 2015 Jan 15;5:786. doi: 10.3389/fmicb.2014.00786 (PMC4295551; doi:10.3389/fmicb.2014.00786)

Figure A4

NanoSIMS images of ^{13}C- and ^{15}N-enriched cells sampled on day 42. (A) Biomass signal represented by $^{12}C^{14}N$ counts, (B) ^{13}C enrichment as determined from the ratio $^{13}C/^{12}C$, (C) ^{15}N enrichment as determined from the ratio $^{12}C^{15}N/^{12}C^{14}N$.

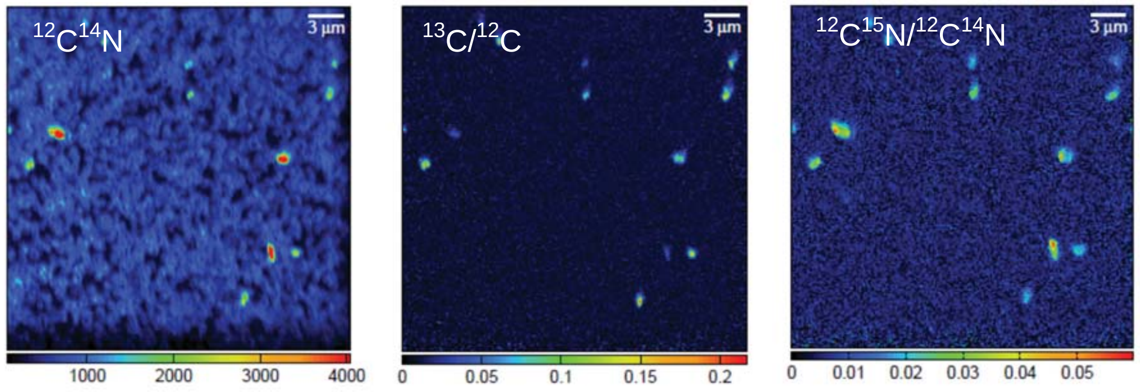

Supplement: Supplementary file 4 [file Image4.PDF]
